# Plasma Homocysteine Level in Children With Postural Tachycardia Syndrome

**DOI:** 10.3389/fped.2018.00375

**Published:** 2018-12-03

**Authors:** Yaqi Li, Bing He, Hongxia Li, Qingyou Zhang, Chaoshu Tang, Junbao Du, Hongfang Jin

**Affiliations:** ^1^Department of Pediatrics, Peking University First Hospital, Beijing, China; ^2^Department of Pediatrics, Renmin Hospital, Wuhan University, Wuhan, China; ^3^Department of Physiology and Pathophysiology, Peking University Health Sciences Center, Beijing, China

**Keywords:** postural tachycardia syndrome, homocysteine, children, heart rate, blood pressure

## Abstract

The study was designed to evaluate the changes of plasma homocysteine (Hcy) level in children with postural tachycardia syndrome (POTS) and explore its significance. A total of 65 subjects were recruited in our study, of whom 35 children were in the POTS group and 30 healthy children were in the control group. Plasma Hcy levels were determined in all subjects. The relationship between the plasma Hcy level and the symptom score was analyzed in the 35 POTS patients. The relationship between the plasma Hcy level and the change in heart rate from the supine to upright position (ΔHR) and between the plasma Hcy level and the rate of increase in heart rate from the supine to upright position (ΔHR/sHR × 100%) were analyzed in all subjects. The plasma Hcy levels were significantly higher in the children with POTS than those in the control group (9.78 [7.68, 15.31] μmol/L vs. 7.79 [7.46, 9.63] μmol/L, *P* < 0.05). The plasma Hcy levels were positively correlated with symptom scores in the POTS patients (*n* = 35, *r* = 0.522, *P* < 0.01). The plasma Hcy levels were also positively correlated with ΔHR (*n* = 65, *r* = 0.332, *P* < 0.01) and ΔHR/sHR × 100% (*n* = 65, *r* = 0.341, *P* < 0.01) in all the subjects. In conclusion, the plasma Hcy levels were elevated in the children with POTS positively correlated with the severity of POTS, suggesting that Hcy might be involved in the pathogenesis of POTS.

## Introduction

Postural tachycardia syndrome (POTS) is a common functional cardiovascular disease in children and adolescents that accounts for ~32.2% of all cases of syncope in children (1–3). Lin et al. (4) had investigated 600 Chinese children and adolescents. Their results showed that the prevalence rate of POTS in Chinese children and adolescents was around 6.8%. The clinical manifestations of POTS are a series of orthostatic intolerance (OI) symptoms, including dizziness, headache, palpitations, chest discomfort, shortness of breath, blurred vision, and sometimes syncope, especially in severely ill patients (5, 6). The haemodynamic feature is an excessive increase in the heart rate (HR) that occurs upon standing, without a substantial change in blood pressure (BP). POTS-related symptoms seriously affect quality of life and impose great burdens on families. Therefore, investigating the pathogenesis of this syndrome and finding effective treatments are necessary. The pathogenesis of POTS is very complicated and has not been studied thoroughly. Previous studies have shown that POTS may be related to autonomic nerve dysfunction, hypovolemia, decreased skeletal muscle pump activity and injured vascular endothelium demonstrated by flow-mediated vasodilation (FMD) and midregional pro-adrenomedullin (MR-proADM) (7–13). Autonomic nerve dysfunction plays an important role in the induction of this disease, and the increased baroreflex sensitivity (BRS) is the key factor (14, 15). BRS has been associated with the severity of POTS and could be used as an effective index to predict the short-term outcome of POTS in children (14). This finding demonstrated the vital role of increased BRS in the mechanism of POTS. However, the reasons for increased BRS in POTS are unclear and require further study. Homocysteine (Hcy), a cytotoxic sulfhydryl-containing amino acid, is an intermediate product in the methionine cycle. Hyperhomocysteinemia (HHcy) is thought to be an independent risk factor for many cardiovascular diseases (CVDs) (16). Possible mechanisms by which Hcy causes CVD include effects on autonomic nerve function, increased oxidative stress, promotion of inflammation, proliferation of smooth muscle cells, and injury to vascular endothelium (16, 17). A study of hypertensive patients found that patients with higher Hcy levels showed substantially higher BRS than those with relatively lower Hcy levels (18). Resstel et al also found that BRS and BP were increased in rats with higher Hcy levels compared to those in the control group (19). Moreover, along with the decrease in Hcy levels, BRS and BP were recovered (19). The results suggested the likelihood that Hcy increased BRS, thereby being involved in the pathogenesis of hypertension. Similarly, Hcy may be the reason for increased BRS in POTS patients. Therefore, we determined plasma Hcy levels in children with POTS and analyzed the correlation between the Hcy levels and the severity of POTS to evaluate the role of Hcy in the pathogenesis of POTS.

## Methods

The subjects included 35 children with POTS (15 males and 20 females), all of whom were admitted to the Department of Pediatrics at Peking University First Hospital due to OI symptoms between February 2015 and January 2018. The age range was 6~13 years with a median of 11 years. All the patients met the criteria for POTS including: (1) mainly elder children; (2) with OI symptoms, such as dizziness and headache, palpitation, nausea, blurred vision and even syncope; (3) positive head-up tilt test (HUTT) or standing-up test; and (4) other diseases that could cause orthostatic intolerance symptoms, including cerebral vascular diseases and organic heart diseases were excluded (20, 21). The control group included 30 healthy children (12 males and 18 females) without a history of syncope or presyncope as demonstrated by history-taking, physical examination and laboratory tests. The age range was 9~12 years with a median of 10 years. All the children, including the control subjects, met the following inclusion criteria: normal physical examination results, routine biochemistry results, and absence of chronic organic diseases including hypertension, seizure, hypothyroidism, anemia and renal dysfunction. We also excluded children with a body mass index (BMI)>24 kg/m^2^ because obesity and lack of exercise could influence Hcy levels. We obtained approval from the Ethics Committee of Peking University First Hospital for the study and fully informed all guardians of the purpose and methods of the study. The written informed consents were obtained from the guardians before our study began.

Symptom scoring was applied to evaluate the severity of POTS (13, 22). Scores were calculated according to occurrence frequency of clinical symptoms. Clinical symptoms included syncope, dizziness, chest tightness, nausea, palpitation, headache, blurred vision, hand shaking and cold sweats. Scoring was as follows: 0, no symptoms; 1, symptoms less than once per month on average; 2, symptoms 2–4 times per month on average; 3, symptoms 2–7 times per week on average; and 4, symptoms more than once per day on average. All the scores consisted of the sum of each symptom-based score.

The protocol for standing-up test or HUTT was according to the previously published literatures (20, 21). For HUTT, children were required to discontinue all drugs that might affect autonomic nervous system function 3 days prior to testing. Children fasted 12 h before the test. The test was performed in a quiet environment with dim light at a comfortable temperature. First, children laid on the tilt table, and HR, BP and electrocardiogram (ECG) recordings were performed for at least 10 min. Then, they were tilted upward at an angle of 60° for 45 min, with simultaneous monitoring of HR, BP and EEG (Dash 2000 Monitor, GE Company, U.S.A.). The patients were placed in the supine position (from the standing position) as soon as a positive response or symptoms of OI occurred. These symptoms included dizziness, headache, fatigue, blurred vision, chest tightness, palpitation, hand tremor, intolerance to movement, and even syncope. Standing-up test was also performed in a comfortable and quiet environment. First, children were laid supine for at least 10 min and HR, BP and ECG recordings were performed. Then, they were tilted upward at an angle of 90° for 10 min, with simultaneous monitoring of HR, BP and EEG (Dash 2000 Monitor, GE Company, U.S.A).

When a child with clinical symptoms of OI had an HR increase ≥40 beats/min or an HR of ≥130 bpm (children aged 6–12 years) or ≥125 bpm (adolescents aged 13–18 years) during the initial 10 min of HUTT or standing-up test, without orthostatic hypotension (a BP decrease>20/10 mmHg), the result was defined as a positive response, and a diagnosis of POTS was made (20, 23).

The change in HR from the supine to upright position (ΔHR) was calculated as the maximum upright HR (uHR) during the initial 10 min minus the mean supine HR (sHR). The rate of the HR increase from the supine to upright position was calculated by dividing ΔHR by the sHR and multiplying by 100 to express the result as a percentage (ΔHR/sHR × 100%). These two indices relatively objectively reflected the severity of POTS.

To determine the plasma Hcy level, a sample with 4 ml of venous blood was obtained from the forearm of each subject. Plasma samples were separated within 30 min of collection. Plasma Hcy was measured using an auto-biochemical analyser (AU5800, Beckman Coulter Company, U.S.A.) by the enzymatic method. This method uses the S-adenosylhomocysteine (SAH) hydrolase reaction principle, in which SAH is hydrolysed by hydrolytic enzymes into adenosine and Hcy; adenosine is immediately hydrolysed into ammonia and hypoxanthine, and nicotinamide adenine dinucleotide (NADH) is converted to NAD by ammonia and glutamic dehydrogenase. The concentration of Hcy in the sample is proportional to the NADH transformation rate. The detection reagents were provided by DiaSys Diagnosis System GmbH (Shanghai) Co., Ltd.

All statistical analyses were performed using the SPSS 20.0 Statistical Package Program for Windows (SPSS, Chicago, IL, U.S.A.). Comparisons of gender (male/female ratio) between the POTS group and the control group were performed by the chi-square test. Measurement data are presented as the mean and SD. Comparisons of supine systolic BP (sSBP) and supine diastolic BP (sDBP) between the POTS group and the control group were performed using an independent Student's *t*-test. Age, BMI, supine HR (sHR) and plasma Hcy levels are expressed as medians and quartiles. Comparisons between the POTS group and the control group were performed using the Mann-Whitney U test. Spearman correlation analysis was used for the correlation analysis. A value of *P* < 0.05 indicated a significant difference.

## Results

No significant differences were found between the two groups in terms of gender, age, BMI, sHR, sSBP, or sDBP (*P* > 0.05; Table 1).

**Table 1:** Baseline characteristics and plasma Hcy levels of the study subjects.

**Characteristics**	**POTS group**	**Control group**	***P*-value**
Cases, *n*	35	30	-
Males/females, n	15/20	12/18	0.816
Age, year	11.00 (8.00, 12.00)	10.00 (9.75, 10.25)	0.080
BMI, kg/m^2^	17.13 (16.14, 18.55)	16.81 (15.15, 19.25)	0.585
sHR, bpm	85.00 (80.00, 90.00)	81.00 (76.05, 87.00)	0.184
sSBP, mmHg	106.09 ± 9.09	108.47 ± 7.36	0.255
sDBP, mmHg	62.31 ± 7.58	64.57 ± 8.66	0.268
Plasma Hcy, μmol/L	9.78 (7.68,15.31)	7.79 (7.46,9.63)	0.013^*^

*Values represent n, mean SD, or median (interquartile range). BMI, body mass index; Hcy, homocysteine; POTS, postural tachycardia syndrome; sHR, supine heart rate; sSBP, supine systolic blood pressure; sDBP, supine diastolic blood pressure*;

* *P < 0.05 vs. the control group*.

**Figure 1:**
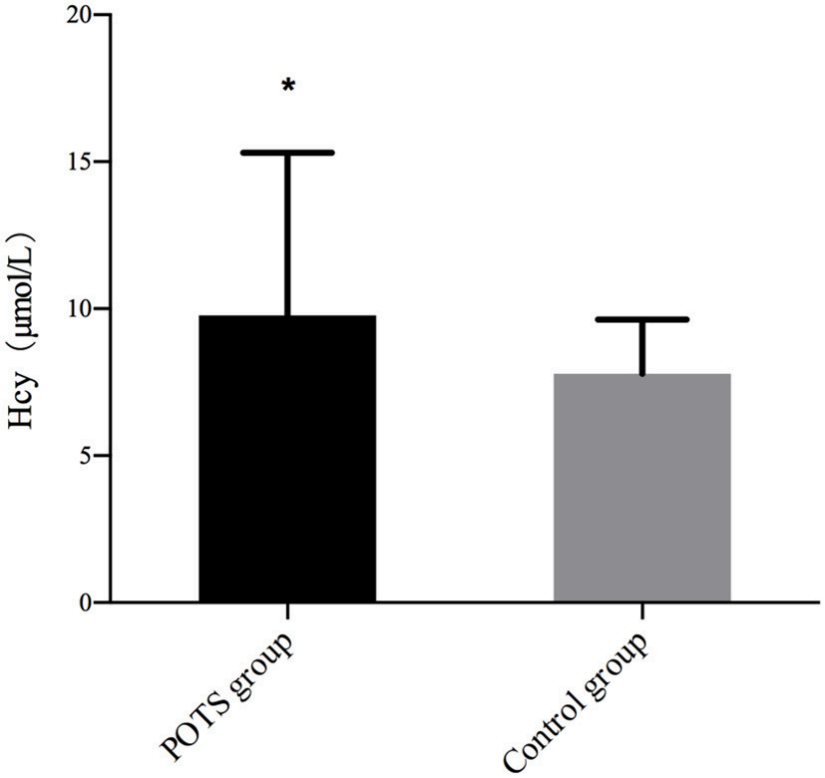
Comparison of plasma Hcy levels between the two groups. The plasma Hcy levels were significantly higher in the POTS group than those in the control group (9.78 [7.68, 15.31] μmol/L vs. 7.79 [7.46, 9.63] μmol/L, *P* < 0.05). Hcy, homocysteine; POTS, postural tachycardia syndrome; *vs. Control group.

However, the plasma Hcy levels were significantly higher in the POTS group than those in the control group (9.78 [7.68, 15.31] μmol/L vs. 7.79 [7.46, 9.63] μmol/L, *P* < 0.05; Table 1, Figure 1).

**Figure 2:**
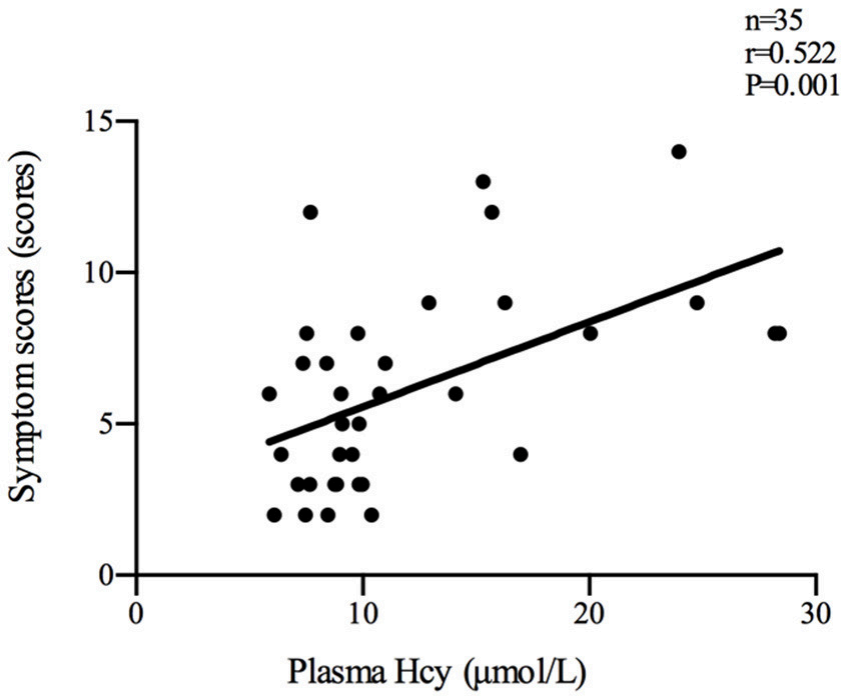
Relationship between plasma Hcy levels and symptom scores. Plasma Hcy levels were positively correlated with the symptom scores of POTS patients (*n* = 35, *r* = 0.522, *P* < 0.01). Hcy, homocysteine; POTS, postural tachycardia syndrome.

**Figure 3:**
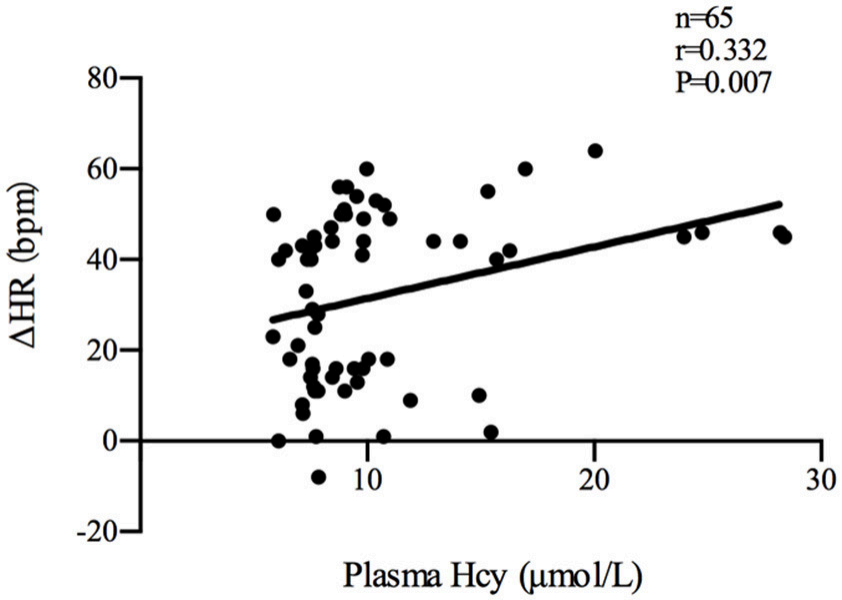
Relationship between plasma Hcy levels and ΔHR. Plasma Hcy levels were positively correlated with the ΔHR (*n* = 65, *r* = 0.332, *P* < 0.01) in all the subjects. ΔHR: change in heart rate from the supine to upright position; Hcy, homocysteine; POTS, postural tachycardia syndrome.

**Figure 4:**
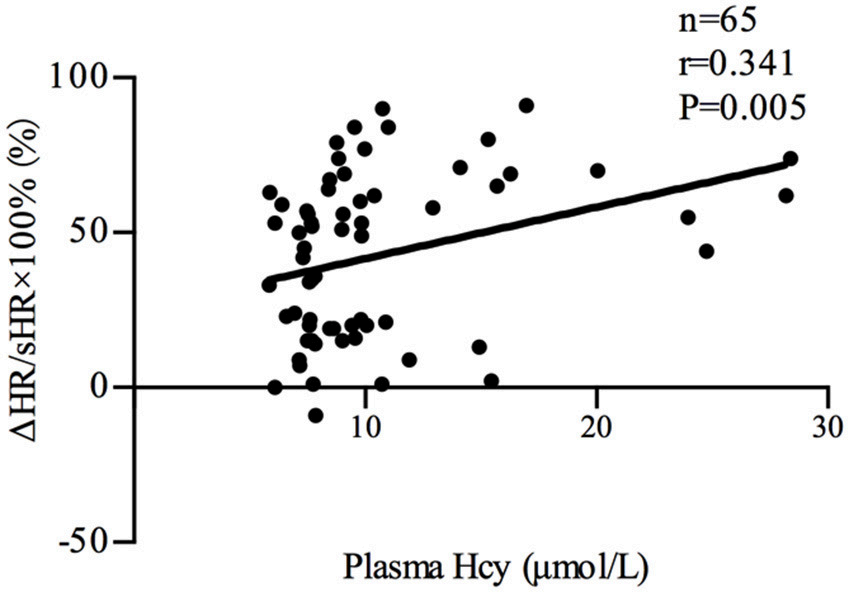
Relationship between plasma Hcy levels and ΔHR/sHR × 100%. Plasma Hcy levels were positively correlated with ΔHR/sHR × 100% (*n* = 65, *r* = 0.341, *P* < 0.01) in all the subjects. ΔHR/sHR × 100%: heart rate increase from the supine to upright position; Hcy, homocysteine; POTS, postural tachycardia syndrome.

Plasma Hcy levels were positively correlated with the symptom scores of POTS patients (*n* = 35, *r* = 0.522, *P* < 0.01; Figure 2). Plasma Hcy levels were also positively correlated with the ΔHR (*n* = 65, *r* = 0.332, *P* < 0.01; Figure 3) and ΔHR/sHR × 100% (*n* = 65, *r* = 0.341, *P* < 0.01; Figure 4) in all the subjects.

## Discussion

POTS is a class of diseases with similar clinical features and haemodynamic changes and is the main cause of OI symptoms in children (1, 21). Approximately 30~40% of children with POTS cannot attend school regularly, which negatively impacts their lives and education (24). To date, the exact mechanisms of POTS remain unclear, and the therapies are also unsatisfactory. Therefore, further studies of the pathogenesis of POTS and the search for effective treatments are particularly urgent.

Muenter Swift et al reported that POTS patients had exaggerated muscle sympathetic nerve activity (MSNA) responses to baroreflex challenges (including vasoactive drug boluses, Valsalva maneuvers and the standing-up test) compared to healthy control subjects (15). Li et al. also found that BRS in children with POTS was significantly higher than that in healthy children (18.76 ± 9.96 ms/mmHg vs. 10 ± 5.42 ms/mmHg, *P* < 0.01) (14). BRS was positively correlated with HR changes in the POTS group (*r* = 0.304, *P* < 0.05), indicating that BRS was related to the severity of POTS. They also performed a 90-day clinical follow-up of patients using conventional therapy (including oral rehydration saline, autonomic nervous function exercises, and health education). They showed that BRS was higher in the non-effective group than that in the effective group (24.7 ± 9.9 ms/mmHg vs. 13.5 ± 6.6 ms/mmHg, *P* < 0.01) and that the use of a BRS value of 17.01 ms/mmHg as a cut-off yielded a sensitivity of 85.7% and specificity of 87.5% for prediction of the short-term outcome of POTS in children (14). These studies suggested that BRS was increased in patients with POTS and that this increased BRS closely influences the development and outcome of the disease. When the body changes to an upright position or is tilted up from a supine position, the returned blood volume decreases, and thus, cardiac output decreases. Correspondingly, local BP decreases, stimulating the carotid sinus and aortic arch baroreceptor to transmit nerve impulses acting on the vasomotor center, reflexively causing an increase in HR and peripheral vascular contraction to maintain normal BP. However, POTS patients are highly sensitive to changes in local BP due to increased BRS, causing an over-increment of the HR in the process.

The previous study found that plasma Hcy levels were significantly higher in the POTS group than those in the control group (9.78 [7.68, 15.31] μmol/L vs 7.79 [7.46, 9.63] μmol/L, *P* < 0.05). In addition, plasma Hcy levels were positively correlated with symptom scores in the POTS patients (*n* = 35, *r* = 0.522, *P* < 0.01). Plasma Hcy levels were also positively correlated with ΔHR (*n* = 65, *r* = 0.332, *P* < 0.01) and ΔHR/sHR × 100% (*n* = 65, *r* = 0.341, *P* < 0.01) in all the subjects. Symptom scores, ΔHR and ΔHR/sHR × 100% are important indicators that reflect the severity of POTS. As a result, not only an increase in plasma Hcy levels was observed in children with POTS but also plasma Hcy levels were significantly related to the severity of POTS, indicating that Hcy may be involved in the pathogenesis of POTS.

As a cytotoxic sulfhydryl-containing amino acid, Hcy is closely associated with various CVDs (16). Tayama et al found that BRS was higher in hypertensive patients with high Hcy levels than that in hypertensive patients with relatively low Hcy levels (18). Resstel et al established an animal model with high Hcy by feeding rats DL-homocysteine thiolactone (DL-HT) (19). The HR and BP were significantly increased in rats with high Hcy levels. A more substantial increment in HR was noted after administering sodium nitrate to the high Hcy group compared to that in the control group; this result reflected the increased BRS in the high Hcy group. In addition, the elevated HR, BP and BRS recovered with the decline in plasma Hcy after discontinuation of DL-HT (19). The results suggested that the increase in Hcy led to increased BRS, and after lowering Hcy levels, a corresponding decrease in BRS occurred.

Since our results showed that plasma Hcy levels were significantly higher in children with POTS and that the levels were closely associated with the severity of POTS, we inferred that Hcy might be involved in the mechanism of POTS by increasing BRS.

The present study has limitations, including a relatively small number of patients, which might have led to bias. We did not examine the reasons for elevated plasma Hcy levels in children with POTS, including the lack of folic acid and vitamin B_12_, decreases in the activity of enzymes that metabolize Hcy or the mutation of gene-encoding enzymes. Further studies are needed to overcome these shortcomings.

Despite these limitations, to the best of our knowledge, this is the first study of changes and the clinical value of plasma Hcy levels in children with POTS, which provides the basis for further exploration of the pathogenesis of POTS. Many studies have shown that folic acid and vitamin B_12_ reduce the risk of CVD by reducing Hcy levels (25–27). As we found that Hcy levels were closely related to the severity of POTS, we hypothesize that a benefit may be obtained from reducing Hcy levels using folic acid and vitamin B_12_. This provides a new approach for effective treatment of POTS, requiring further study.

## Author contributions

YL and BH contributed to the design of the study, literature overview, data analysis, and preparation of the first draft of the manuscript. HL contributed to sample collection, data acquisition and data analysis. QZ contributed to clinical diagnosis and reviewed the manuscript. CT contributed to review and correction of the manuscript. JD and HJ contributed to manuscript design, clinical diagnosis and final review and correction of the manuscript. All authors have read and approved the final manuscript.

### Conflict of interest statement

The authors declare that the research was conducted in the absence of any commercial or financial relationships that could be construed as a potential conflict of interest.
